# Supplementary material for: Inclusion of chia seeds (Salvia hispanica L.) and pumpkin seeds (Cucurbita moschata) in dairy sheep diets
Source: PLoS One. 2024 Apr 18;19(4):e0300864. doi: 10.1371/journal.pone.0300864 (PMC11025919; doi:10.1371/journal.pone.0300864)
Supplement: Attachment — Submitted filename: editorial observations evbp.docx [file pone.0300864.s002.docx]

1. Please note that funding information should not appear in the Acknowledgments section/Funding section or Any other areas of your Manuscript. We will only publish funding information present in the Funding Statement section of the online submission form. Please remove any funding-related text from the manuscript.

AUTHORS: The accepted protocol for animal care and use for this study is the same as the one reported for funding. It is a MUST to declare the Id PROTOCOL used for the study and in our Institution is the same project code for funding or animal use and care approval.

2. We note that the grant information you provided in the 'Funding Information' and 'Financial Disclosure' sections do not match.

AUTHORS: This has been revised and made it match accordingly.

When you resubmit, please ensure that you provide the correct grant numbers for the awards you received for your study in the 'Funding Information' section.

AUTHORS: This has been revised and made it match accordingly.

3. Thank you for updating your data availability statement. You note that your data are available within the Supporting Information files, but no such files have been included with your submission. At this time we ask that you please upload your minimal data set as a Supporting Information file, or to a public repository such as Figshare or Dryad.

Please also ensure that when you upload your file you include separate captions for your supplementary files at the end of your manuscript.
AUTHORS: we have uploaded our data at FIGSHARE: 10.6084/m9.figshare.24424525

As soon as you confirm the location of the data underlying your findings, we will be able to proceed with the review of your submission.
