# Supplementary material for: Inclusion of chia seeds (Salvia hispanica L.) and pumpkin seeds (Cucurbita moschata) in dairy sheep diets
Source: PLoS One. 2024 Apr 18;19(4):e0300864. doi: 10.1371/journal.pone.0300864 (PMC11025919; doi:10.1371/journal.pone.0300864)
Supplement: Attachment — Submitted filename: REBUTTAL LETTER PlosOne.docx [file pone.0300864.s003.docx]

REBUTTAL LETTER

Dear Authors

Although you have addressed major issues of the current manuscript, however, there are few changes especially grammar and some numerical values need to be addressed. Authors need these to be addressed before publication. Authors are advised to respond and address the said comments

AUTHORS

We have made English style corrections and double-checked numbers. All changes have been done in red colour.
